# Prevalence of HPV high and low risk types in cervical samples from the Italian general population: a population based study

**DOI:** 10.1186/1471-2334-10-214

**Published:** 2010-07-20

**Authors:** Paolo Giorgi Rossi, Simonetta Bisanzi, Irene Paganini, Angela Di Iasi, Claudio Angeloni, Aurora Scalisi, Rosalba Macis, Maria Teresa Pini, Francesco Chini, Francesca Maria Carozzi

**Affiliations:** 1 Laziosanità - Agency for Public Health, Lazio Region. Via di S. Costanza 53, 00198 - Rome, Italy; 2 Analytical and Biomolecular Cytology Unit, Cancer Prevention and Research Institute, ISPO, Via Cosimo il Vecchio 2, Florence - 50139, Italy; 3 Anatomia Patologica, Ospedale G. Moscati, ASL Caserta 2, Aversa (CE) - 81021, Italy; 4 Coordinamento Screening, ASL Teramo, Teramo - 64100, Italy; 5 Coordinamento Screening, ASL Catania, Catania - 95124, Italy; 6 Anatomia Patologica, ASL Cagliari 09125, Italy; 7 Coordinamento Screening, Dipartimento Materno Infantile, ASL Napoli 2, Giugliano in Campania (NA) - 80014, Italy

## Abstract

**Background:**

This multicenter study describes the type-specific prevalence of HPV infection in the general population from central and southern Italy, comparing the data with previously published Italian studies.

**Methods:**

Women aged from 25 to 65 who attended cervical cancer screening in five different Italian regions were tested for HPV infection with Hybrid Capture II (HCII) low and high risk probes. Women repeating Pap-test upon unsatisfactory or positive results, or as a post-treatment and post-colposcopy follow-up analysis, were excluded from our study. High risk (HR) HPV positive samples were typed using GP5+/GP6+ primed PCR, followed by Reverse Line Blot for 18 high/intermediate risk HPV types, while low risk (LR) HPV positive samples were tested with type specific primers for HPV6 and HPV11.

**Results:**

3817 women had a valid HCII test: 350 of them (9.2%) were positive for HR probes, 160 (4.2%) for LR probes, while 57 women were positive for both. Multiple infections were detected in 97 HR HPV positive women. The most common types were HPV 16 (3%), 31 (1.2%), 51 (1%). HPV6 ranked fifth (0.6%), HPV18 ranked tenth (0.5%) and HPV11 sixteenth (0.3%).

In Sardinia the prevalence of high-risk infection was 13%, significantly higher than the mean value (p < 0.00005).

The distribution of the most frequent types did not significantly differ by centre (p = 0.187) and age (p = 0.085).

**Conclusions:**

Because cervical cancer incidence and Pap test coverage is lower in southern than in northern Italy, a lower prevalence of high-risk infections in the general population was expected in the south. However, prevalence detected in this study for the south of the country is slightly but significantly higher than the rest of Italy. The consequence may be an epidemic of cervical cancer in the next decades if adequate screening programs are not implemented there.

## Background

The identification of HPV as a necessary cause of cervical cancer [1] has been rapidly followed by the introduction of new tools for prevention: HPV test and vaccine.

In Italy the vaccine has been recommended, free and offered to 11-year old girls since the beginning of 2008 [2].

### Pre-vaccination campaign studies

In 2006, the Italian Centre for Disease Control and Prevention planned a set of studies to assess HPV epidemiology before introducing the vaccine. The main aims of these studies were: to describe the prevalence of types in the healthy population and in women affected by cervical neoplasia, to monitor any type replacement in vaccinated cohorts, to assess the proportion of infections by HPV strains present in the vaccine, to estimate the cost effectiveness of various vaccine and screening strategies, and to describe the knowledge about the virus and attitudes towards HPV vaccination in young women.

In particular, detection of HPV types in the healthy population was performed in three different studies, focusing on the following populations:

• the NTCC (New Technologies in Cervical Cancer) biobank, which included all HPV positive samples from about 50.000 women aged between 25 and 60 in central and northern Italy, excluding Rome [3-5]. They were tested for HR HPV types;

• a large sample of women aged between 18 and 26, newly recruited across Italy (sample size: 4000 women). They underwent HPV test and typing;

• a large sample of women aged between 25 and 65, newly recruited in Rome and southern Italy (sample size: 4000 women). They underwent HPV test and typing and are the object of the present report.

### Previous knowledge on HPV epidemiology in Italy

Previously published Italian studies of circulating HPV types in healthy women were affected by two main problems: small sample size and limited extension of the analysed geographical area. Most of them were performed on samples from asymptomatic women who had spontaneously turned to gynaecologic clinics for cervical cancer prevention or reproductive problems [6,7]. Only two studies were based on a predetermined population sample. In these cases, women were recruited from small geographical areas [8,9] and one study was focused on young women only [9].

In southern Italy, the epidemiology of cervical cancer is different compared to the rest of the country (table 1) [10]. In particular, incidence is slightly lower than in the centre and north. On the other hand, Pap test coverage is higher in the north than in the south [11]. The combination of these two data suggests differences in the underlying HPV epidemiology.

**Table 1:** Cervical Cancer incidence, survival, Pap test coverage and Extension of Cervical cancer Programmes by geographical area.

	North West	North East	Centre	South and Island	Italy
Incidence/100.000 2001-05 (AIRTum) [10]	7.1		7.3	6.2	7
Incidence/100.000 1996-2000 (AIRTum) [10]	8.5		7.5	7.4	8.1
Survival 2001-05 [10]	65%		68%	59%	65%
Pap test coverage (ISTAT 2004-05) [11]	79.5%	85.1%	78.1%	52.3%	70.9%
Cervical Cancer Screening Program implementation (GISCi) 2001 [12]	59.8%		68.8%	23.5%	40.1%
Cervical Cancer Screening Program implementation (GISCi) 2007 [12]	65.4%		91.9%	68.7%	71.8%

AIRTum: Associazione Italiana Registri Tumori; ISTAT: Istituto Nazionale di Statistica; GISCi: Gruppo Italiano Screening del Cervicocarcinoma

### Aim of the study

The aim of the present study was to measure the prevalence of different HPV types in the general population attending population-based screening programmes in Rome and southern Italy and to compare the data with previously published Italian studies.

## Methods

### Setting

The study was conducted within cervical cancer screening programmes that actively invite the entire target population (age ranging from 25 to 64) in Rome and southern Italy. A convenience sample of the existing screening programmes was selected: in Sicily and Sardinia only two programmes issued invitations regularly and were therefore selected, in Molise, Apulia and Calabria there was no consolidated programme when the study started and no data were collected from these regions, in Abruzzo it was possible to collect samples from the whole region, while in Rome metropolitan area and Campania, the most populated areas, we selected two programmes each.

In 2007, the target population for cervical cancer screening, i.e. women aged 25-64, included 5.71 million units in southern Italy and 1.18 million units in the metropolitan area of Rome. The active screening programmes in these areas actually covered 4.75 million women (69%) [12], while the target population of the programs included in the sample was 1.50 million women (22% of the total target population, 32% of the population living in an area with an active screening program).

### Population and sample size

During the study period the programme invited the target population, without any selection criteria (nor age neither previous screening participation). We later sampled about 800 women in each region (planned sample size was 5 × 800, 4000). 25 to 65 year old women were included in our study. Those repeating Pap-test upon unsatisfactory or positive results, or as a post-treatment and post-colposcopy follow-up, were excluded.

Sample size (4000 women), was chosen in order to achieve the two following objectives:

1.+/-3% precision in the estimate of the proportion of infections of a type responsible for at least 10% of infections;

2. 95% power to see any type responsible for at least 1% of infections.

Considering overall HPV prevalence to be 10%, we expected 400 samples would be HPV positive and therefore available for typing.

### Sample collection

Cervical scrape samples were collected in ThinPrep vials containing PreservCyt (Cytyc Corp., Marlborough, USA) transport medium or in Specimens Transport Medium (STM) (DNAPAP cervical sampler, Qiagen, Gaithersburg, USA). Before testing, 400 μl of STM samples, vortexed on a shaking platform at 1000 rpm for at least 10 minutes, were biobanked at -80°C.

### HPV testing

The presence of high risk (HR) and low risk (LR) HPVs in cervical specimens was evaluated by Hybrid Capture II^® ^(HCII) (Qiagen, Gaithersburg, USA) [12-17] using probemix B, specific for 13 HR HPV types: 16, 18, 31, 33, 35, 39, 45, 51, 52, 56, 58, 59 and 68, and probemix A, specific for 5 LR HPV types: 6, 11, 42, 43 and 44. To lyse cells and release DNA, specimens collected in PreservCyt^® ^(no more than 20 samples at a time) were processed with Digene Sample Conversion kit (Qiagen, Gaithersburg, USA), according to the manufacturer's instructions. HCII assay followed. HPV DNA testing was done locally in 3 sites (one in Abruzzo and two in Rome) while samples collected in Cagliari and Catania were analysed in Florence (ISPO). Results of HCII tests for HPV DNA were expressed as relative light units (RLU): specifically, the ratio of the specimen light emission to the average emission of three concurrently tested positive controls (1 pg/ml HPV DNA). All samples with a ratio value over 1 were considered HPV positive. HCII quality assurance procedures were performed as previously described [18].

An additional internal control was then used in each HCII test plate. At the beginning of the study, each laboratory denatured a quality control LR HPV sample (HPV type 6 DNA) and a quality control HR HPV sample (HPV type 16 DNA) provided with the kit. 100 μl of denaturated control sample were aliquoted in 15 tubes and stored at -20°C. Single aliquots were then used in every HCII plate. Quality controls for both LR and HR HPV had a specific concentration of virus DNA (5 pg/ml) and therefore an expected ratio value ranging from 2 to 8.

For external quality assurance, 22 STM samples (100 μl) were sent to one of the laboratories involved in the study (Florence, ISPO) and samples had to be tested twice. Laboratories that used PreservCyt^® ^medium, sent 15 additional ThinPrep samples (4 ml) to the same facility, where the whole HCII procedure was repeated.

Results were expressed as specimens in RLU/CO; a modal value (i.e. the most frequently positive or negative value reported) was defined for each case in the external control set and considered as the reference result. The result provided from each single laboratory was compared with the modal value.

### Typing procedures

Typing procedures were centralized in Florence at the ISPO laboratory and all the centres involved in the study sent HR or LR HPV positive samples there.

For DNA extraction, 1.5 ml of samples in PreservCyt^® ^solution and 200 μl of STM samples were used to collect DNA with a QIAamp DNAMini Kit. Samples were eluted in 100 μl AE buffer preheated at 70°C. An HPV negative sample was included in each batch of extraction to exclude any contamination during this phase.

HCII HR HPV positive specimens were amplified and typed with "consensus High Risk HPV genotyping kit" (Qiagen); this system uses PCR with biotinilated GP5+/GP6+ primers, followed by reverse Line Blot for 18 high risk HPV types: 16, 18, 26, 31, 33, 35, 39, 45, 51, 52, 53, 56, 58, 59, 66, 68, 73 and 82.

PCR was performed in 50 μl final volume with 10 μl DNA, 5 μl 10× buffer II, 7 μl 25 mM MgCl_2 _, 10 μl 1 mM dNTPs, 0,3 μl AmpliTaqGold (Applied Biosystem) DNA polymerase (5 U/μl) and 2 μl of Qiagen Primer set, as recommended by the manufacturer. The thermal cycler program contained ramping times between the temperature used for denaturation, annealing and elongation that appeared essential for optimal performance [19]. Activation of the Taq polymerase (9 min at 94°C) was followed by 40 PCR cycles: denaturation at 94°C for 20 seconds, with 2,8°C/sec ramp speed, annealing at 38°C for 30 seconds, with 1,8°C/sec ramp speed, and elongation at 71°C for 80 seconds with 1,8°C/sec ramp speed, and a final extension at 71°C for 4 minutes [19]. Amplified products were run on 2% agarose gels containing ethidium bromide and visualized by ultraviolet light.

All samples, independently from gel results, underwent reverse line blot analysis. Biotinylated PCR products were hybridized with specific oligonucleotide probes bound to membrane strips. After stringent washing, streptavidin-conjugated alkaline phosphatase was added and a non-radioactive reaction performed using a chromogenic substrate. Strips containing the resulting purple precipitates were analysed visually from an interpretation grid supplied with the kit. A biotinylated poly(dT) control for conjugate reaction is present in each strip to ensure good test performance and a proper alignment of the strips on the interpretation sheet. Samples were considered positive for a specific HPV type if the precipitate was viewed in the specific position of the strip. In each run we included two negative controls (a purified HPV negative DNA sample extracted in the same batch as the test ones, and a DNA-free sample) and two positive controls (HPV16 plasmid purchased with the kit and a known non-16 HR HPV DNA).

GP5+/6+PCR-negative and reverse Line Blot-negative samples were amplified for the β-globin gene using primers GH20-PC04 (268 bp amplicon lenght) to assess the integrity of DNA [20].

β-globin positive samples were re-typed with "INNO-LiPA HPV genotyping Extra Amp" (Innogenetics, Ghant, Belgium) following the manufacturer's instructions, because this system can detect 28 HPV HR or LR types: 6, 11, 16, 18, 26, 31, 33, 35, 39, 40, 43, 44, 45, 51, 52, 53, 54, 56, 58, 59, 66, 68, 69, 70, 71, 73, 74 and 82. The β-globin negative samples were extracted and typed again.

The remaining un-typed samples (GP5+/6+PCR-positive, reverse Line Blot-negative and INNO-LiPA negative) were considered HPV X type.

HCII LR HPV positive specimens (probe A specific for 5 LR types: 6,11,42,43,44) were typed using HPV 6 and HPV 11 specific primers and resulting negative samples were considered positive for the other LR types included in probemix A (HPV 42,43,44). No LR type, other than 6 and 11, was searched for.

Primer sequences for HPV 6 were 5'-TAGTGGGCCTATGGCTCGTC-3' (sense) and 5'-TCCATTAGCCTCCACGGGTG-3' (antisense) which amplified a 289 bp product.

Primer sequences for HPV 11 were 5'-GGAATACATGCGCCATGTGG-3' (sense) and 5'-CGAGCAGACGTCCGTCCTCG-3' (antisense) which amplified a 360 bp fragment [21].

PCR reactions were performed in 25 μl reaction volume using 5 μl DNA, 2,5 μl 10× buffer II, 1,5 μl 25 mM MgCl_2_, 2 μl 2,5 mM dNTPs, 1,25 μl 10 μM primers, 0,2 μl Taq Gold (Applied Biosystems). Hot-starting at 94°C for 6 min was followed by 40 cycles as follows: 95°C for 30 sec, 58°C for 30 sec, 72°c for 30 sec and an hold at 72°C for 5 min. Amplified products were run on 2% agarose gels containing ethidium bromide and visualized by ultraviolet light.

### Systematic review

We searched the Medline using the terms ("HPV" OR ("human" AND "Papillomavirus")) AND ("type" OR "Typing") AND ("Italy" OR "Italian") AND ("cervix" OR "cervical") AND "prevalence". The search was updated on 15/12/2009. We excluded all studies that selected the pathological population, women in follow-up or with a positive Pap test. We included only studies reporting prevalence in the general healthy population. We extracted the total HPV positivity, the positivity to high risk HPV, the proportion of HPV16 among isolated HPV, the proportion of HPV 18 and the proportion of cases with HPV16 or 18, the age of the population, the geographical area.

### Analysis

The following analyses were planned a priori in the study design: prevalence of HPV positivity to high and low risk probes by age, by centre; prevalence of HPV 16-18 by age, by centre; proportion of HPV types by age, by centre. We calculated 95% confidence intervals taking into account the two stages sample using STATA 8 survey module [22].

### Ethics

The study has been approved by the Ethics Committee of the Istituto Superiore di Sanità (the Italian National Institute of Health, CE-ISS 07-162 e 07/163). Women were informed of an additional HPV test in the screening procedure and asked to sign a consent form.

## Results

### Overall prevalence

In this study, 3817 women had one valid sample for HPV testing, with both HR and LR probes. The overall positivity was 9.2% (350/3817) (95% CI 6.2-12.2) for high and 4.2% (160/3817) (95% CI 2.3-6.1) for low risk types.

The prevalence of cytological modification was 3% of ASCUS or more severe, in the centres using the Pap test as primary screening.

**Figure 1:**
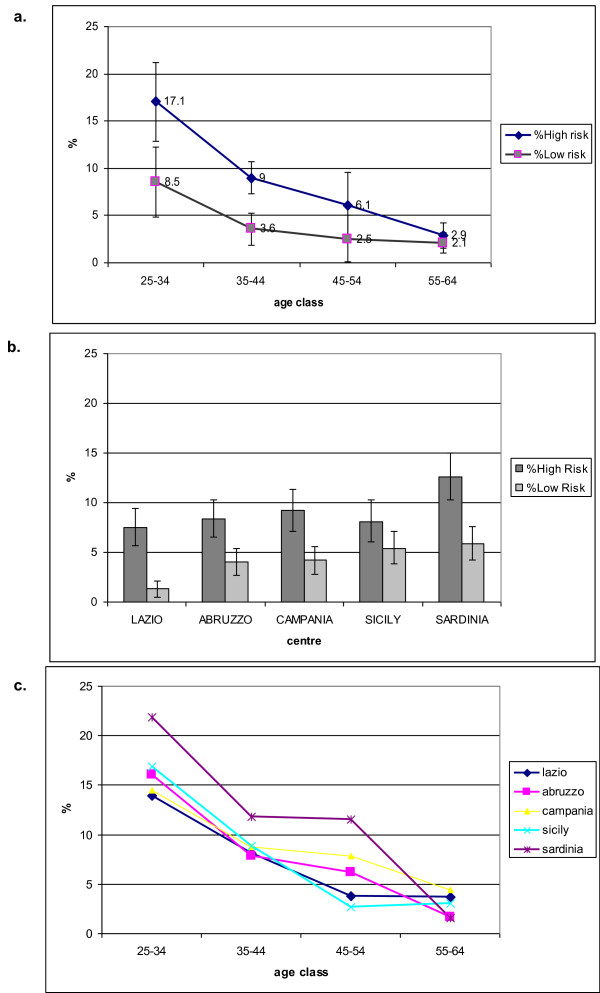
**Prevalence of HPV infection by type and age (a), type and centre (b), and age and centre, High Risk only (c)**.

Figure 1a shows the prevalence of HR and LR type infection by age. Both are monotone decreasing curves, but the slope is steeper for HR (slope -4.6 p < 0.00005) than LR prevalence (slope -2.1 p < 0.00005). The two slopes significantly differ (test for parallel slopes p < 0.00005), consequently the ratio between HR and LR prevalent infections is 2, i.e. 17.1% (148/866) and 8.5% (74/866), for 25-34 year old women and 1.4, i.e.2.9% (15/517) and 2.1% (11/517), in 55-64 year old women.

The overall prevalence varies significantly by centre (p < 0.00005 for HR and p < 0.00005 for LR types). The observed variability is mostly due to a higher prevalence of HR HPVs in Cagliari (Sardinia) and to a lower prevalence of LR types in Rome (Figure 1b).

The age-specific prevalence curves do not show heterogeneity except in Cagliari (Sardinia) that has a steeper negative slope of the curve (test for parallel slopes p < 0.00005) (Figure 1c).

The HCII quality control included co-testing of synthetic samples with known HPV DNA concentration and the circulation of clinical samples between laboratories to assess reproducibility, which was found to be very high (kappa coefficient = 0.93 for positive vs. negative).

### Typing

Samples that showed high and/or low risk HPV with HCII were 449: 293 among them displayed infection by HR HPVs only, 99 by LR HPVs only, while 57 by both HR and LR HPVs. In women younger than 25, we detected 12 infections: 6 HR HPVs only, 4 LR HPVs only and 2 HR and LR HPVs. These cases were excluded from the analyses.

Out of 350 HR HPV positive samples, 300 (85.7%) were immediately typed using the "Consensus High Risk genotyping HPV" (Qiagen), 15 (4.3%) were typed with INNO-LiPA "HPV Genotyping Extra Amp" (Innogenetics), 12 (3.4%) samples resulted PCR positive but remained un-typed with reverse line blot Digene and INNO-LiPA (10 HR only and 2 HR + LR) and the remaining 23 (6.6%) beta-globin positive samples were classified as HPV negative samples. Co-infections were present in 97 HR positive women (27.7% among positive samples, 2.5% of the general population).

Out of 160 LR HPV identified by probemix A HCII (HPV 6, 11, 42, 43, 44), four samples were not available for typing, 24 (15.4%) samples resulted HPV 6, 10 samples (6.4%) were positive for HPV 11 (4 of them were HPV 6 and 11 co-infections) and 126 samples (80.7%) were classified positive for other LR HPV types. No other specific LR type was searched for.

The most common types were 16 (2.8%), 31 (1.1%), 51 (0.8%), 66 (0.7%) and 6 and 56 (0.6% both); HPV18 ranked tenth (0.5%) and HPV11 ranked sixteenth (0.3%). Other low and undertermined risk types were found for a total of 31 different types, some of these were not included in the HCII probemixes. This fact is due to cross-hybridization. Previous studies, in fact, suggested that HCII using probemix B may cross-react with HPV types either not associated or with undetermined associations with cancer (non-oncogenic HPV) [23].

Table 2 shows HPV types by regional occurrence. While HPV 16 is the most frequent type in all centres, the second most frequent type is different in the various centres: it is HPV 31 in Rome, Abruzzo and Campania and it changes on the islands, HPV 6 in Sicily (p < 0.00005) and HPV 51 in Sardinia (p = 0.0025).

**Table 2:** Distribution of HPV types by age and centres

		**Rome**		**Campania**		**Abruzzo**		**Sicily**		**Sardinia**		**All**	
		
		**N**	**%**	**N**	**%**	**N**	**%**	**N**	**%**	**N**	**%**	**N**	**%**
		
**All age**		803	100.0	755	100	800	100.0	782	100	727	100	3867	100
	<25*	*1*	*0.1*	*31*	*4.1*	*0*	*0.0*	*5*	*0.6*	*9*	*1.2*	*46*	*1.2*
	25-34	144	17.9	173	22.9	181	22.6	130	16.6	238	32.7	866	22.4
	35-44	258	32.1	241	31.9	255	31.9	295	37.7	245	33.7	1294	33.5
	45-54	265	33.0	241	31.9	241	30.1	221	28.3	173	23.8	1141	29.5
	55-64	134	16.7	67	8.9	123	15.4	130	16.6	62	8.5	516	13.3
	65+*	*1*	*0.1*	*2*	*0.3*	*0*	*0.0*	*1*	*0.1*	*0*	*0.0*	*4*	*0.1*
**25-64 years**		**801**		**722**		**800**		**776**		**718**		**3817**	
	**HR+ only**	55	6.9	61	8.4	51	6.4	47	6.1	79	11.0	293	7.7
	**LR+ only**	9	1.1	25	3.5	17	2.1	27	3.5	25	3.5	103	2.7
	**HR & LR+**	1	0.1	7	1.0	15	1.9	11	1.4	23	3.2	57	1.5
**types**													
	**6**	1	0.1	2	0.3	5	0.6	9	1.2	7	1.0	24	0.6
	**11**	2	0.2	1	0.1	4	0.5	0	0.0	4	0.6	11	0.3
	**16**	17	2.1	19	2.6	23	2.9	20	2.6	29	4.0	108	2.8
	**18**	5	0.6	2	0.3	3	0.4	1	0.1	7	1.0	18	0.5
	**26**	2	0.2	1	0.1	1	0.1	0	0.0	0	0.0	4	0.1
	**31**	11	1.4	7	1.0	10	1.3	4	0.5	9	1.3	41	1.1
	**33**	4	0.5	5	0.7	1	0.1	3	0.4	6	0.8	19	0.5
	**35**	5	0.6	1	0.1	0	0.0	7	0.9	5	0.7	18	0.5
	**39**	2	0.2	1	0.1	4	0.5	4	0.5	6	0.8	17	0.4
	**40**	0	0.0	1	0.1	3	0.4	0	0.0	0	0.0	4	0.1
	**42**	0	0.0	5	0.7	4	0.5	0	0.0	0	0.0	9	0.2
	**43**	0	0.0	1	0.1	4	0.5	0	0.0	0	0.0	5	0.1
	**44**	0	0.0	0	0.0	0	0.0	1	0.1	0	0.0	1	0.0
	**45**	5	0.6	1	0.1	6	0.8	0	0.0	2	0.3	14	0.4
	**51**	4	0.5	3	0.4	2	0.3	6	0.8	17	2.4	32	0.8
	**52**	4	0.5	4	0.6	3	0.4	5	0.6	7	1.0	23	0.6
	**53**	1	0.1	4	0.6	1	0.1	5	0.6	10	1.4	21	0.6
	**54**	0	0.0	1	0.1	1	0.1	0	0.0	0	0.0	2	0.1
	**56**	3	0.4	4	0.6	4	0.5	2	0.3	11	1.5	24	0.6
	**58**	4	0.5	5	0.7	2	0.3	5	0.6	6	0.8	22	0.6
	**59**	1	0.1	0	0.0	4	0.5	5	0.6	4	0.6	14	0.4
	**66**	3	0.4	5	0.7	4	0.5	4	0.5	10	1.4	26	0.7
	**68**	0	0.0	2	0.3	5	0.6	1	0.1	3	0.4	11	0.3
	**70**	1	0.1	1	0.1	1	0.1	1	0.1	1	0.1	5	0.1
	**73**	1	0.1	2	0.3	1	0.1	0	0.0	3	0.4	7	0.2
	**81**	0	0.0	1	0.1	2	0.3	0	0.0	0	0.0	3	0.1
	**82**	0	0.0	1	0.1	1	0.1	1	0.1	1	0.1	4	0.1
**not typed**													
	**HR+**	0	0.0	22	3.0	2	0.3	4	0.5	6	0.8	34	0.9
	**LR+**	7	0.9	14	1.9	6	0.8	19	2.4	21	2.9	67	1.8
**co-infections HR**													
	**2**	9	1.1	11	1.5	15	1.9	19	2.4	26	3.6	80	2.1
	**3**	3	0.4	5	0.7	0	0.0	1	0.1	6	0.8	15	0.4
	**4**	1	0.1	0	0.0	0	0.0	0	0.0	1	0.1	2	0.1

*not included in the analyses

HR: High Risk; LR: Low Risk

Table 3 shows the types by age. No evident trend can be seen for any single virus type, but the prevalence of HPV 16 is lower in 55-65 year old women, even if not significantly.

**Table 3:** Distribution of HPV positivity and types by age

		<25*		**25-34**		**35-44**		**45-54**		**55-64**		**All§**	
		
		***N***	***%***	**N**	**%**	**N**	**%**	**N**	**%**	**N**	**%**	**N**	**%**
		
**All**		*46*	*100.0*	866	100	1294	100	1141	100	516	100	3817	100
	**HR+ only**	*6*	*13.0*	108	12.5	105	8.1	67	5.9	13	2.5	293	7.7
	**LR+ only**	*4*	*8.7*	34	3.9	34	2.6	26	2.3	9	1.7	103	2.7
	**HR & LR+**	*2*	*4.3*	40	4.6	12	0.9	3	0.3	2	0.4	57	1.5
**types**													
	**6**	*0*	*0.0*	15	1.7	4	0.3	2	0.2	3	0.6	24	0.6
	**11**	*0*	*0.0*	2	0.2	3	0.2	4	0.4	2	0.4	11	0.3
	**16**	*5*	*10.9*	48	5.5	37	2.9	21	1.8	2	0.4	108	2.8
	**18**	*1*	*2.2*	12	1.4	4	0.3	0	0.0	2	0.4	18	0.5
	**26**	*0*	*0.0*	2	0.2	1	0.1	0	0.0	1	0.2	4	0.1
	**31**	*2*	*4.3*	15	1.7	15	1.2	6	0.5	5	1.0	41	1.1
	**33**	*1*	*2.2*	8	0.9	8	0.6	3	0.3	0	0.0	19	0.5
	**35**	*0*	*0.0*	7	0.8	5	0.4	4	0.4	2	0.4	18	0.5
	**39**	*0*	*0.0*	7	0.8	8	0.6	2	0.2	0	0.0	17	0.4
	**40**	*0*	*0.0*	2	0.2	1	0.1	1	0.1	0	0.0	4	0.1
	**42**	*1*	*2.2*	5	0.6	3	0.2	1	0.1	0	0.0	9	0.2
	**43**	*0*	*0.0*	4	0.5	1	0.1	0	0.0	0	0.0	5	0.1
	**44**	*0*	*0.0*	0	0.0	1	0.1	0	0.0	0	0.0	1	0.0
	**45**	*0*	*0.0*	3	0.3	4	0.3	6	0.5	1	0.2	14	0.4
	**51**	*2*	*4.3*	15	1.7	10	0.8	6	0.5	1	0.2	32	0.8
	**52**	*0*	*0.0*	11	1.3	8	0.6	4	0.4	0	0.0	23	0.6
	**53**	*0*	*0.0*	11	1.3	5	0.4	4	0.4	1	0.2	21	0.6
	**54**	*0*	*0.0*	0	0.0	0	0.0	1	0.1	1	0.2	2	0.1
	**56**	*0*	*0.0*	10	1.2	7	0.5	7	0.6	0	0.0	24	0.6
	**58**	*0*	*0.0*	8	0.9	10	0.8	3	0.3	1	0.2	22	0.6
	**59**	*0*	*0.0*	6	0.7	7	0.5	3	0.3	0	0.0	16	0.4
	**66**	*1*	*2.2*	11	1.3	12	0.9	1	0.1	0	0.0	24	0.6
	**68**	*0*	*0.0*	5	0.6	4	0.3	2	0.2	0	0.0	11	0.3
	**70**	*1*	*2.2*	1	0.1	2	0.2	2	0.2	0	0.0	5	0.1
	**73**	*0*	*0.0*	6	0.7	0	0.0	1	0.1	0	0.0	7	0.2
	**81**	*0*	*0.0*	0	0.0	1	0.1	1	0.1	1	0.2	3	0.1
	**82**	*0*	*0.0*	1	0.1	1	0.1	2	0.2	0	0.0	4	0.1
**not typed**													
	**HR+**	*1*	*2.2*	11	1.3	8	0.6	11	1.0	4	0.8	34	0.9
	**LR+**	2	*4.3*	21	2.4	23	1.8	17	1.5	6	1.2	67	1.8
**co-infections**													
	**2 types**	*3*	*6.5*	39	4.5	26	2.0	10	0.9	5	1.0	80	2.1
	**3 types**	*0*	*0.0*	6	0.7	6	0.5	2	0.2	1	0.2	15	0.4
	**4 types**	*1*	*2.2*	1	0.1	1	0.1	0	0.0	0	0.0	2	0.1

* these cases are excluded from analyses; four women over 65 were all negative for HPV

§ excluding <25 years.

HR: High Risk; LR: Low Risk

Out of 97 women with HR HPV co-infections, 43 (44.3%) included HPV 16 and/or HPV 18. Table 4 shows the matrix of co-infections.

**Table 4:** Matrix of co-infections in women positive to High Risk probe.

	**HPV type**																		
	
**HPV type**	**18**	**26**	**31**	**33**	**35**	**39**	**40**	**44**	**45**	**51**	**52**	**53**	**56**	**58**	**59**	**66**	**68**	**70**	**73**
**16**	6	2	3	6	5	5	2		3	4	4	4	2	2		5	1	1	1
**31**		1			1	1				1	1			1		2			2
**33**		2								2		1	1		1	2			
**35**	2														1	2			
**39**	1	1	1		1										1	1			
**42**			2							1									1
**43**			1						1										
**45**	1		1		1														
**51**	1										1	3	1	1			1		
**52**					1					1					1	1	1		1
**53**			1														1		
**54**			1									1							
**56**				1						1	2	1			1				
**58**				1	1	1						1	1			1			
**66**	1									1					2		1		
**70**								1		1	1								
**81**			1																
**82**										1			1			1			1

Triple infections are reported as three couples, quadruple infections as 12 couples. N 97

note:15 triple infection: 18/16/35 two; 16/66/33; 16/31/35; 31/73/42; 16/31/45; 16/33/51; 54/31/53; 51/52/56; 16/51/56; 16/53/58; 33/56/58; 35/59/66; 51/66/68; 16/52/70

2 quadruple infections 16/39/18/66; 16/26/31/39

We have information on 17 histologically-confirmed CIN2 or more severe lesions found among HPV positive women. Including three co-infections, in these samples we detected 21 types: the most frequent types were 16 (12 cases) followed by HPV types 31, 35, 66 (2 cases each) and HPV types 33, 58, 73 (1 case each).

### Systematic review

We found 72 papers in the PubMed search. Eleven of them fulfilled the inclusion criteria, two more papers were later excluded thorough analysis because prevalence data on HPV types were available only for a subset of the healthy population selected according to Pap test finding. Further literature was found in the IARC meta-analysis: none of these papers were eligible because they investigated only HR HPV prevalence and did not report any typing.

Table 5 summarises results from the analysed studies. The overall prevalence, in the same age classes, is lowest in population-based studies [8,9], medium in opportunistic screening studies [6,7] and highest in gynaecological clinic-based studies [24,25,27,28,30,31]. The two studies based on migrant population [26,29] showed the highest overall prevalence and most widespread composition of types.

**Table 5:** Systematic review of the published studies on prevalence of HPV types in the general population in Italy

							percentage of positive in the population						
									
Author	pub year	Area	N	Age	Population	type of test	HR+LR	HR	HPV16	HPV18	HPV16+18	Notes on molecular methods	Notes on possible selection biases
Astori [24]	1997	Udine	197	mean 37.7	women referred to gynaecological clinic for genital complaints, Pap negative	PCR My09/11 and RFLP analysis	20.3		5.1				
Ronco [8]	2005	Turin	1000	25-70	Invited in Organized Screening program	PCR-EIA Gp5+/Gp6+ home made	8.8	7.1	2.8	0.1	2.9		
Centurioni [6]	2005	Genoa	503	mean 51	Opportunistic screening	PCR Dot blot	15.9		9.1	5.8	14.9	the method is not actual any more	The recruitment started in 1992
Tornesello [25]	2006	Milan and Neaples	207	median 40	asymptomatic attending clinic	PCR home made and direct sequence	19.7	16.9	8.7	0	8.7		
Tornesello [26]	2007	Neaples	31	mean 28.9	migrant	PCR nested (MY09-11 follwed by the inner GP5+/6+)followed by sequencing	35.5	25.8	6.4	0	6.4		
Ammatuna [9]	2008	Sicily	1006	18-24	random population sample	Linear array HPV genotyping test (Roche)	24.1	17.4	4.5	1.3	5.8		
Tornesello [7]	2008	Neaples	115	mean 32.5	Opportunistic screening	Home made Nested PCR followe by direct sequencing	13.9	13.9	6.9	0.9	7.8		
Del Prete [27]	2008	Apulia	871	>20; 85% = <50	women referred to hospital gynecologic visit	PCR E1-E2 followed by gel	23.1	19.4	9.6	1.6	10.1		Not clear reason for referral, includes women referred for colposcopy
Agarossi [28]	2009	Italy	9947	15->65; 85% <50	asymptomatic attending clinic	HCII followed by typing with Gp5+/Gp6+ PCR EIA Home made		14.8	4.3	1.5	5.6	no mention of negative control for PCR	the sample includes women with previous positive Pap test
Giovannelli [29]	2009	Sicily	120	88% <40	Migrant attending Clinical visit	PCR-Inno-LiPA	47.8	34.8	7.8	6	13.8		
Masia [30]	2009	Sardinia	815	>18; 83% = <45	Counselling for sexual activity or screening program	PCR-EIA Gp5+/Gp6+ home made	31	20.3	8.2		13.5		
Verteramo [31]	2009	Rome	857	17-57	asymptomatic attending clinic	PCR MY09/11 (and PCR with 4 pairs of primers to amplifiy E6 and E7 gene) followed by sequencing	31	13.3	4.4	0.8	5.3		

HPV 16 prevalence ranges from 2.8% to 9.6%; again the lowest prevalence is registered in population-based studies. HPV 18 prevalence ranges from 0 to 6%.

**Figure 2:**
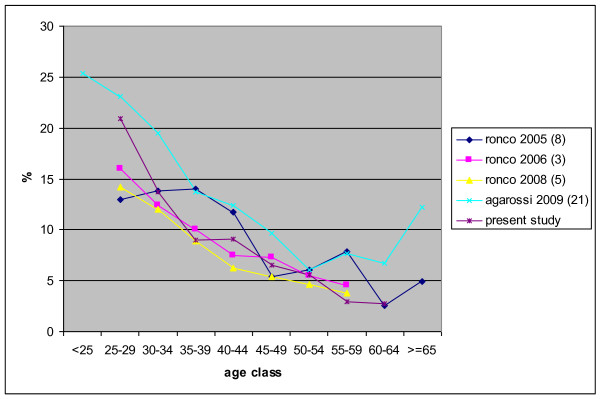
**High risk HPV prevalence of infection by age in the Italian studies published until now**.

Among included and excluded studies, only 4 were large enough to give age-specific prevalence of HR-HPV. Figure 2 reports prevalence by age.

## Discussion

### HR HPV prevalence

The overall prevalence observed in our study is lower than what observed in most of the previous Italian studies even if we consider the same age classes (table 5). All previous studies but two [8,9] were performed on a population attending gynaecological clinics, including opportunistic screening, or on high-risk populations. In these studies, the observed prevalence of lesions is quite high. Our study, in measuring HR HPV prevalence, has an identical design and identical molecular methods of the NTCC trial. Notably, results are extremely similar, even if the centres included are not the same and the geographical areas have social, cultural, economical and historical differences. We will explore the implications of this finding in the next paragraph. A more detailed comparison is required from the recent study by Agarossi and coll[28]. This study has a very large sample with a quite low prevalence of ASCUS+ cytological modification (6.4%). In our study, the prevalence of cytological modification was 3%. Sampling women who visit gynaecological clinics may select those who are more sexually active than the average population. Furthermore, having a previous abnormal Pap test increases the probability of turning to the clinic for further analyses, as noted by Agarossi and coll. [28]. On the contrary, screening programmes included in our study invite all the target population; furthermore, in our programme all women not visiting the clinic for a new first level round were excluded from the analyses. Figure 2 compares the curves of HR HPV prevalence of infection by age in the Italian studies published until now: they show a similar decreasing curve of prevalence at younger ages. Not surprisingly, studies based on organised screening programmes show a lower prevalence than the study based on the population attending gynaecological clinics [28], particularly at older ages.

HPV 16 prevalence has a similar variability than the overall HPV prevalence, reflecting the same difference due to the study-base. The variability in HPV 18 prevalence is higher; this difference may reflect the stronger influence of random fluctuations for this quite rare type. Not surprisingly the two studies on migrant population show a higher prevalence and a wider spectrum of types [26,29]. In fact, migrant women attending gynaecological clinics are strongly selected to be at high risk for sexually transmitted diseases.

In our study, we observed a strong variability among centres in the circulating types, with the exception of HPV 16. Most of the differences are due to Cagliari (Sardinia). This area is relatively isolated, compared with other Italian regions, as supported by observations on genetic background [35].

### LR HPV prevalence

The ratio between high and low risk infection types decreases with age. This finding has been observed in other studies [34]. There are several possible explanations for this phenomenon and unfortunately a cross sectional study cannot disentangle the question: 1) different types may circulate in different cohorts and there may be little exchange between the two; 2) LR and HR types may elicit different immune responses and the pool of immunised women for some of the more frequent high risk viruses may increase in older cohorts, consequently the prevalence of high risk infections decreases more rapidly with age due to a more rapid decrease in the susceptible population for high than low risk viruses; 3) low risk infections, even while having virtually no probability of progression, may also have lower probability of clearance in the long term.

### The relation between HPV prevalence and cervical cancer incidence

The incidence of invasive cancer in Italy is slightly lower in the South, 6.2/100.000, than in the North and Centre, 7.1/100.000 and 7.3/100.000 respectively [10], despite a much lower Pap test coverage in 2004-05, according to the National Health Interview [11]: 83%, 78% and 52%, in the North, Centre, South respectively. The two observations, i.e. a slightly lower incidence and a much lower Pap test coverage in the south, had been interpreted until now as indirect evidence that HPV was less diffuse in this area and as indicative of different sexual behaviours. Now we have studies allowing for comparison of HPV prevalence in different Italian areas: the overall prevalence observed in our study in southern Italy is very similar to that observed in the NTCC study in central and northern Italy, even if slightly higher [5]. More precisely, prevalence is higher only in younger women, while in women aged 55 or older it is slightly lower. This finding is consistent with the study by Agarossi and coll. [28] that did not find any geographical trend. In the last decades, rapid changes in sexual behaviours occurred in Italy, particularly in younger people. It is plausible that the cultural and behavioural changes occurred earlier in northern than in southern Italy.

In our opinion, lower prevalence of HPV infection in the previous decades is still the only plausible explanation for lower incidence of invasive cancer in the South. In fact the incidence currently observed by cancer registries is the consequence of infections acquired several years ago. In this scenario, the slightly higher prevalence of infection observed now in southern compared to in northern Italy, particularly in younger cohorts, may indicate that the South is in transition from low to medium prevalence of HPV. As a result, we may expect an increase in invasive cervical cancer incidence in the next decades if a better control by screening programme is not implemented. This interpretation is coherent with the cervical cancer incidence trends estimated by De Angelis and coll. [32], predicting differences between North and South would decrease.

Actually, the implementation of cervical cancer screening in southern Italy is going on, even if with great difficulties, and the effect on Pap test coverage in some areas is now detectable [33].

### Limits

We tested women who were invited and participated to cervical cancer screening programmes in Rome and southern Italy. Response to organised screening is quite low, ranging from 25 to 50% in the selected areas; the low participation may introduce a self-selection bias. Nevertheless, active invitation was the only means we had of reducing self-selection. Previous studies found few socio-economic differences between participants and not participants [36,37].

A second limit, due to the nesting of this study into organised screening programmes, is that we could not include some regions, such as Calabria and Apulia, because at the time the study started there were no active programmes that contacted the whole target population.

We decided not to gather information on the cytology results in recruited women, since one centre used HPV as the primary screening test. This choice limited the opportunity of analyses.

We typed only women positive to the HR and LR HCII probes; according to this protocol we may have missed women who are positive to other HPV types not included in the probes or samples with very low number of virus DNA copies.

## Conclusions

Pap-test coverage in southern Italy is lower than in central and northern Italy. Nevertheless cancer registries until 2003 showed a similar or even lower cervical cancer incidence in southern Italy than the rest of the country. In this situation, a lower prevalence of HPV infection in southern Italy was expected. Actually, the observed HPV prevalence in the general female population is slightly higher, in particular in younger cohorts.

We interpret this finding as indicative of a transition in southern Italy, from a low to medium-high prevalence area.

The final effect of this transition may be an epidemic of cancer in the next decades, if appropriate screening programmes are not implemented to control the disease.

## Abbreviations

HPV: human papillomavirus; HR HPV: high risk human papillomavirus types; LR HPV: low risk human papillomavirus types; PCR: polymerase chain reaction; HCII: Hybrid Capture II ^®^; RLU/CO: Relative light units/cut-off; STM: standard transport medium; NTCC: New Technologies in Cervical Cancer; ASCUS+: Atypical Squamous Cells of Undetermined Significance or more severe cytology

## Competing interests

Financial: PGR received travel reimbursement for two international conferences by Sanofi Pasteur MSD; FMC is occasional advisor to Gen-Probe, Abbot, Sanofi and GlaxoSmithKline.

Non financial: PGR is employed in the HTA Unit of the Agency for Public Health of the Lazio Region, this Agency contributes in establishing public screening programs.

## Authors' contributions

PGR designed and conducted the study and drafted the paper; FC designed the laboratory procedures, coordinated the laboratory quality control and contributed in drafting the paper; SB and IP performed the typing test; FC designed the data flow, managed the data and performed the statistical analyses; AS, CA, RM, MTP, ADI coordinated the study in the recruiting centres. The Working Group contributed in recruiting the women, performing the HPV tests, collecting the data.

All the authors approved the final version of the paper.

## Pre-publication history

The pre-publication history for this paper can be accessed here:

http://www.biomedcentral.com/1471-2334/10/214/prepub
